# What does “Advanced” mean in 2023? reflecting on 10 years of the ESTRO advanced Skills in modern radiotherapy course

**DOI:** 10.1016/j.tipsro.2023.100227

**Published:** 2023-12-01

**Authors:** E. Forde, M. Josipovic, M. Kamphuis, J. Lopez, P. Remeijer, S. Rivera, P. Scherer, L. Wiersema, R. de Jong

**Affiliations:** a Applied Radiation Therapy Trinity (ARTT), Discipline of Radiation Therapy, School of Medicine, Trinity St. James's Cancer Institute, Trinity College Dublin, University of Dublin, Dublin, Ireland; b Department of Clinical Medicine, Faculty of Health, University of Copenhagen, Copenhagen Denmark; c Medical Imaging and Radiation Therapy, Inholland University of applied sciences, Haarlem, the Netherlands; d Department of Radiation Oncology, Instituto de Biomedicina de Sevilla/Hospital Universitario Virgen del Rocío/CSIC/Universidad de Sevilla, Sevilla, Spain; e Department of Radiation Oncology, The Dutch Cancer Institute, Antoni van Leeuwenhoek, Amsterdam, Netherlands; f Institut Gustave-Roussy, Villejuif, France; g University Clinic for Radiotherapy and RadioOncology of the PMU at the County Hospital Salzburg, Austria; h Department of Radiation Oncology, Amsterdam University Medical Centres - location AMC, Cancer Institute Amsterdam, Amsterdam, Netherlands

**Keywords:** ESTRO School, Education, Advanced Skills

## Abstract

The roles and responsibilities of radiation therapists (RTTs) are many and varied. Professional expectations are influenced by the technology available, as well as the level of autonomy RTTs have in their daily practice. This professional range requires RTTs to possess a unique set of ever evolving skills, posing challenges from an educational perspective.

Teaching these “advanced skills” has been the ambition the ESTRO Advanced Skills in Modern Radiotherapy course. In the 10th year of this course, the Faculty look back and reflect on how our programme has evolved and what it has achieved.

## Highlights

• The roles and responsibilities of RTTs are many and varied.
• Teaching “advanced skills” to a diverse audience is challenging.
• The ESTRO ASMR course meets these challenges with a dynamic programme.

## Background

It’s obvious to state that radiotherapy has advanced significantly over the past 10 years; driven largely by increased biological insights and technological innovations in our field. What is less obvious is how we define “advanced” when referring to the skill set required of radiation therapists (RTTs) using these technologies [1]. Equally difficult to articulate is what we mean by “modern” radiotherapy, especially when patterns of practice vary so greatly between institutions and countries [2], [3].

Describing these terms and maintaining a programme that is both relevant and inclusive for all professions has been an annual challenge faced by the faculty of the ESTRO Advanced Skills in Modern Radiotherapy (ASMR) course. As we find ourselves 10 years older (and arguably wiser), the ESTRO ASMR faculty look back and reflect on what our course has achieved since its inception in 2013.

## Discussion

*The changing Landscape of our participants*

**Fig. 1:**
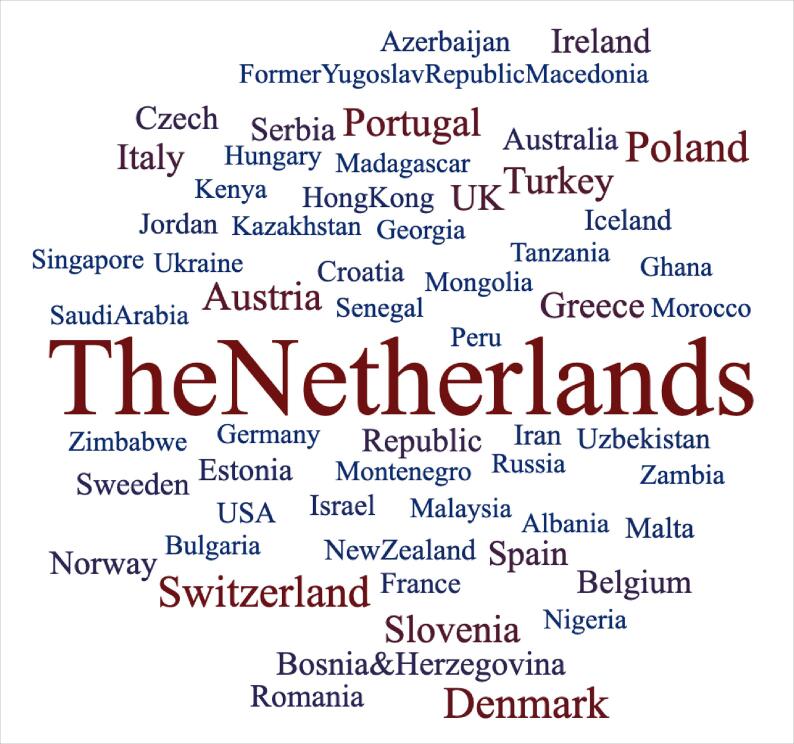
Word cloud representing countries of work of participants from all courses combined. The countries of greater prominence in the word cloud reflect the higher number of participants from those countries.

Over the years, we have taught our programme to over 350 radiotherapy professionals across approximately 58 different countries (Fig. 1). Our first course was held in Amsterdam and unsurprisingly, nearly half of our participants that year were Dutch. Whilst they continue to represent a large percentage of our participant group each year, in 2023 we found ourselves working with over 60 colleagues from 38 different countries. It is with thanks to the support of the International Atomic Energy Agency, we are now reaching previously under-represented African nations, as well as our neighbours in Eastern Europe. This level of heterogeneity may present challenges from a teaching standpoint; however, it has enabled everyone to appreciate the diversity of our international workforce. Roles and responsibilities of RTT can vary greatly even at a European level, and understanding this perspective is critical if the “aspiration of the free movement of radiation therapists in the European Union” is to become a reality [4].

Not only have the geographical origins of our participants changed over the years, but so too has their professional background (Table 1). The ASMR course was conceived to meet the unique educational and professional needs of RTTs, and to provide such a course within the ESTRO School portfolio. We envisaged a participant group made up exclusively of RTTs to deliver a dedicated programme tackling topics which directly support RTTs in their daily practice. Topics which ranged from nuisances in image matching protocols to managing patient side effects. However, RTTs do not work in siloed environments, and given the interdisciplinarity of modern radiotherapy, our course now welcomes all members of the radiotherapy team. This professional diversity has been particularly valuable during the course workshops; for example, during the image guided radiotherapy (IGRT) workshop where radiation oncologists work alongside RTTs discussing image matching and communicating their priorities without the time pressure of having a patient on the treatment couch. Each year we have also had vendor representatives registered as participants. Their engagement, specifically during the failure mode effects analysis (FMEA) workshops, have elevated discussions and provided a unique opportunity for RTTs to communicate their professional perspectives directly with industry partners.

**Table 1:** Demographic information regarding participant’s speciality and years of experience. Data regarding participant speciality and years of experience are based on participants who completed the course feedback survey and may not represent the full cohort of course participants.

Location	Number of participants registered	Participant’s Years of Experience		Participant's Speciality									
		Median (Years)	Range (Years)	RTT (%)	RTT Trainee (%)	RO (%)	RO Trainee (%)	MP (%)	MP Trainee (%)	RB (%)	RB Trainee (%)	Other (%)	Missing Data (%)
2014 Amsterdam	69	10	1 – 26	88.33	6.67	0.00	0.00	0.00	0.00	0.00	0.00	5.00	0.00
2015Copenhagen	42	9	0 – 37	75.61	4.88	4.88	0.00	4.88	4.88	2.44	0.00	2.44	2.44
2016 Dublin	32	10.5	1 – 28	75.00	3.13	6.25	0.00	6.25	0.00	0.00	0.00	9.38	0.00
2017 Prague	34	6	0 – 36	72.41	17.24	0.00	0.00	10.34	0.00	0.00	0.00	0.00	0.00
2018 Rome	44	6	1 – 26	69.70	3.03	12.12	9.09	3.03	0.00	0.00	0.00	3.03	0.00
2019 Brussels	44	7	1.5 – 30	70.59	5.88	5.88	0.00	5.88	0.00	0.00	0.00	11.76	0.00
2020 No course (Covid-19)													
2021 No course (Covid-19)													
2022 Seville	39	11	2–––39	79.49	2.56	2.56	2.56	5.13	0.00	2.56	0.00	5.13	0.00
2023 Paris	62	6	1–––28	60.42	12.50	12.50	2.08	6.25	4.17	0.00	0.00	2.08	0.00

What has remained fairly consistent over the years is the range of professionals who are at different stages of their career, which provides ample opportunities for peer teaching and learning amongst the group (Table 1). This was particularly relevant for our course in which there are many opportunities for small group discussions, both formally and informally. Furthermore, this stable range in years of experience demonstrates the motivation of RTTs to engage in continuous education and life-long learning, irrespective of what stage they are at in their careers. This is, perhaps, unsurprising considering the pace at which our field continues to develop.

*The Changing Landscape of our Programme*.

When our course first started it was originally named Advanced Skills for Treatment Delivery (ASTD). After just one year we felt this name did not sufficiently capture the breadth of the roles and responsibilities of RTTs, nor the breadth of our programme, and so it was re-named to what we have today. Our programme was always designed to capture all aspects of the radiotherapy chain from pre-treatment preparation to follow-up care. Whilst these core topics remain, in recent years we have also expanded content specific to online adaptive radiotherapy, MRI guided treatments, surface guidance, and artificial intelligence; reflecting the changing clinical environments we now work in. As a faculty we acknowledge that professional roles vary internationally; however, it is critical for RTTs to understand the whole radiotherapy chain, and the consequences of the clinical decisions they make in daily practice.

As with many ESTRO courses, the ASMR course delivers content through a combination of didactic lectures and a strong integration of practical workshops. The commitment to this model of teaching meant that the course did not run for two years due to the Covid-19 pandemic, where the faculty felt these interactive workshops could not be successfully transferred to an online setting. The specific approach to some of the workshops has evolved over the years however, and a clear example is our Falcon (Fellowship in Anatomic delineation and CONtouring) workshop embedded into the programme. This workshop now incorporates not just manual delineation of common organs at risk, but a “peer review” of those generated with artificial intelligence. This new approach again reflects the recent changes to radiotherapy practice and responsibilities of the RTTs.

## CONCLUSION

The ESTRO ASMR course aims to foster collaboration and knowledge sharing with international colleagues on topics most relevant to RTT’s daily practice. Our faculty may have aged 10 years, but our programme remains as agile and fresh as ever. As a multidisciplinary faculty we relish healthy debates and whilst opinions may vary, a key message that remains steadfast is our prioritisation and investment into the education of RTTs.

## Declaration of Competing Interest

The authors declare that they have no known competing financial interests or personal relationships that could have appeared to influence the work reported in this paper.
